# Impact of phase I metabolism on uptake, oxidative stress and genotoxicity of the emerging mycotoxin alternariol and its monomethyl ether in esophageal cells

**DOI:** 10.1007/s00204-016-1801-0

**Published:** 2016-07-15

**Authors:** Christine Tiessen, Doris Ellmer, Hannes Mikula, Gudrun Pahlke, Benedikt Warth, Helge Gehrke, Kristin Zimmermann, Elke Heiss, Johannes Fröhlich, Doris Marko

**Affiliations:** 1 Department of Food Chemistry and Toxicology, Faculty of Chemistry, University of Vienna, Waehringer Str. 38, 1090 Vienna, Austria; 2 Institute of Applied Synthetic Chemistry, Vienna University of Technology, Getreidemarkt 9/163, 1060 Vienna, Austria; 3 Department of Pharmacognosy, Faculty of Life Sciences, University of Vienna, Althanstrasse 14, 1090 Vienna, Austria

**Keywords:** *Alternaria alternata*, Reactive oxygen species, Topoisomerase inhibition, KYSE510, Human phase I and II metabolism, Mycotoxin conjugates

## Abstract

Studies on the genotoxicity of *Alternaria* mycotoxins focus primarily on the native compounds. Alternariol (AOH) and its methyl ether (AME) have been reported to represent substrates for cytochrome P450 enzymes, generating hydroxylated metabolites. The impact of these phase I metabolites on genotoxicity remains unknown. In the present study, the synthesis and the toxicological effects of the metabolites 4-hydroxy alternariol (4-OH-AOH) and 4-hydroxy alternariol monomethyl ether (4-OH-AME) are presented and compared to the effects of the parent molecules. Although the two phase I metabolites contain a catecholic structure, which is expected to be involved in redox cycling, only 4-OH-AOH increased reactive oxygen species (ROS) in human esophageal cells (KYSE510), 4 times more pronounced than AOH. No ROS induction was observed for 4-OH-AME, although the parent compound showed some minor impact. Under cell-free conditions, both metabolites inhibited topoisomerase II activity comparable to their parent compounds. In KYSE510 cells, both metabolites were found to enhance the level of transient DNA–topoisomerase complexes in the ICE assay. Although the level of ROS was significantly increased by 4-OH-AOH, neither DNA strand breaks nor enhanced levels of formamidopyrimidine-DNA-glycosylase (FPG)-sensitive sites were observed. In contrast, AOH induced significant DNA damage in KYSE510 cells. Less pronounced or even absent effects of hydroxylated metabolites compared to the parent compounds might at least partly be explained by their poor cellular uptake. Glucuronidation as well as sulfation appear to have only a minor influence. Instead, methylation of 4-OH-AOH seems to be the preferred way of metabolism in KYSE510 cells, whereby the toxicological relevance of the methylation product remains to be clarified.

**Electronic supplementary material:**

The online version of this article (doi:10.1007/s00204-016-1801-0) contains supplementary material, which is available to authorized users.

## Introduction

**Fig. 1:**
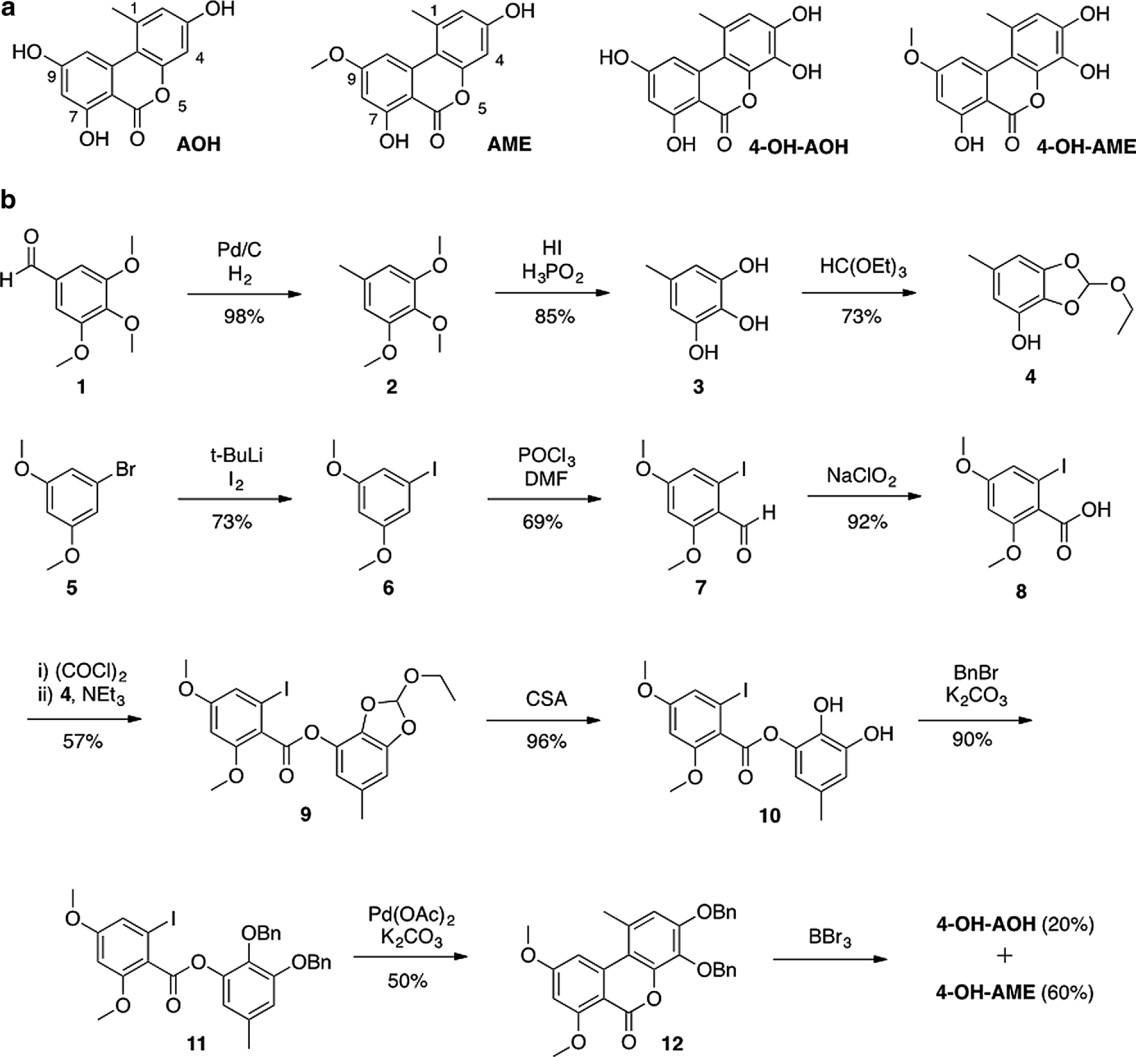
**a** Chemical structure of alternariol (AOH), alternariol monomethyl ether (AME), 4-hydroxy alternariol (4-OH-AOH) and 4-hydroxy alternariol monomethyl ether (4-OH-AME), **b** chemical synthesis of 4-OH-AOH and 4-OH-AME. *Et* ethyl, *t-Bu* tert. Butyl, *DMF* N,N-dimethylformamide, *CSA* camphorsulfonic acid, *Bn* benzyl, *Ac* acetyl

Fungi of the genus *Alternaria* are ubiquitously present in nature, causing a diversity of plant diseases (Mikami et al. 1971; Tsuge et al. 2013). As a result of their wide sporulation and growth range, they infect plants and food crops in nearly every stage of production, even during storage at low temperatures. The excessive production of secondary metabolites by *Alternaria* spp. under diverse conditions enables them to be hazardous to the health of humans and animals (Asam and Rychlik 2013; CONTAM 2011; Lee et al. 2015). Seventy of these secondary metabolites have been classified as mycotoxins or phytotoxins (Barkai-Golan 2008). Alternariol (AOH) and alternariol monomethyl ether (AME) (Fig. 1) represent two of the major mycotoxins produced by *Alternaria alternata* that have been ascribed as cytotoxic and genotoxic in vitro (Pfeiffer et al. 2007a). Fehr et al. (2009, 2010) reported DNA strand-breaking properties of AOH and AME in vitro in consequence of topoisomerase poisoning. Additionally, mutagenic and estrogenic effects in cell culture were described by Lehmann et al. (2006) and Brugger et al. (2006). Some of these activities might be caused by phase I metabolites of AOH and AME. Pfeiffer et al. (2007b) postulated that during xenobiotic metabolism, metabolites of AOH or AME, arising from hydroxylation through cytochrome P450 (CYP) enzymes, have a reactive catechol or hydroquinone structure. Such compounds are supposed to undergo redox cycling inducing the generation of reactive oxygen species potentially leading to DNA damage. Thus, despite data concerning toxicity and other effects of AOH and AME, their phase I metabolites should not be neglected for a proper risk evaluation.

Pfeiffer et al. (2007b, 2008) incubated human microsomes with AOH and AME confirming the formation of metabolites hydroxylated at C-2, C-4 and C-8. Furthermore, CYP1A1, commonly occurring in extrahepatic tissues such as the esophagus (Lechevrel et al. 1999), was the most active monooxygenase for AOH and especially for AME (Pfeiffer et al. 2008; Schreck et al. 2012). Thus, phase I metabolites may be generated in a tissue-specific manner after ingestion of AOH or AME and may at least contribute to the induction of esophageal cancer (Liu et al. 1991). CYP1A1 belongs to the isoenzyme family of CYPs which is mainly regulated by the aryl hydrocarbon receptor (AhR) pathway. As hypothesized by Schreck et al. (2012), AOH and AME are inducers of the AhR pathway, which enhances the expression of several metabolizing enzymes especially CYP monooxygenases. Experiments with murine AhR-deficient Hepa-1c1c12 cells did not show induction of CYP expression after incubation with AOH or AME supporting the hypothesis. Also in line are the findings of Pahlke et al. (2015), who analyzed the impact of *Alternaria* toxins on CYP1A transcription and activity in different human tumor cells with the objective to identify potential organ specificity. AOH caused an induction of CYP1A most prominently in esophageal cells (KYSE510) after 24-h incubation, whereas AME only mediated an increase in liver cells. Because of the enhanced sensitivity of KYSE510 cells toward AOH, the experiments were repeated in AhR-suppressed KYSE510 cells. CYP1A1 induction by AOH was significantly reduced compared to the AhR-expressing cells, but AhR suppression was of no relevance for the DNA-damaging properties of AOH. The data suggest that at least AOH promotes its xenobiotic metabolism by AhR-dependent induction of CYP enzymes in cells.

The lacking impact of AhR suppression on DNA damage might be due to the initiation of cellular defense mechanisms. As recently reported, AME and, to a greater extent, AOH were found to modulate the cellular redox status in human colon cancer cells (HT29), human liver cancer cells (HepG2) and especially KYSE510 cells (Pahlke et al. 2015; Tiessen et al. 2013a). The increase in reactive oxygen species (ROS) results from an imbalance between ROS formation and elimination by scavengers and might on the one hand affect directly DNA, proteins or lipids and on the other hand act as signaling molecules e.g., in the nuclear factor (erythroid-derived 2)-like 2 (Nrf2) pathway. Nrf2 is bound by Kelch-like ECH-associated protein 1 (Keap1) in the cytosolic fraction (Itoh et al. 1999). Electrophiles and ROS provoke the release of the Nrf2 from the Nrf2–Keap1 complex, which can now translocate into the nucleus where it binds in a complex with small Maf proteins to the antioxidant response element (ARE) and initiates the transcription of anti-oxidative enzymes like glutathione-S-transferases (GST), γ-glutamylcysteine synthetases (γ-GCL) or UDP-glucuronosyltransferases (UGT). Furthermore, Miao et al. (2005) reported a direct regulation of Nrf2 by AhR activation leading to an elaborated drug-metabolizing detoxification mechanism made up of phase I and II enzymes. As stated above, the catecholic structure of the phase I metabolites may contribute to the induction of ROS. It can therefore be hypothesized that biotransformation of AOH and AME plays an important role in the severity of the toxic effects. Until now, little is known about the impact of possible metabolites of AOH and AME. It entails the necessity of further investigation of the phase I metabolites to better assess the health risk of *Alternaria* toxins.

In the present study, the question was addressed whether phase I metabolites of AOH and AME with their highly reactive catechol or hydroquinone structure might exceed the toxicological effects of their parent compounds. In a previous study, KYSE510 cells reacted most sensitive toward AOH regarding ROS production and CYP1A induction compared to HepG2 or HT29 cells (Pahlke et al. 2015). Based on these results and a potential link between a high incidence of esophageal cancer in a province of China and the consumption of grains heavily contaminated with *Alternaria* (Liu et al. 1991, 1992), esophageal tumor cells were chosen in this study. In order to compare the effects of the metabolites with those of the parent toxins, the impact on cytotoxicity, induction of ROS generation, topoisomerase targeting, genotoxicity and cellular uptake were investigated in detail.

## Materials and methods

### Chemicals and reagents

AOH and AME were purchased from Sigma-Aldrich Chemie GmbH (Taufkirchen, Germany). 4-OH-AOH and 4-OH-AME were chemically synthesized as shown in Fig. 1. Experimental details are described in the Online Resource 1. The products were purified by semi-preparative HPLC (Knauer, Germany), characterized by NMR spectroscopy (Bruker Avance UltraShield 400, Ettlingen, Germany) and HRMS analysis (Thermo Scientific LTQ Orbitrap XL Hybrid FTMS, Waltham, MA, USA), and their purity (>95 %) was analyzed by HPLC-DAD (Agilent 1200, Waldbronn, Germany) at a wavelength of 340 nm. Etoposide (ETO) was bought from Sigma-Aldrich (Taufkirchen, Germany). Kinetoplast DNA (kDNA) and topoisomerase IIα were obtained from TopoGEN\ (Port Orange, FL, USA). Topoisomerase IIα and IIβ-specific rabbit polyclonal antibodies, specific rabbit polyclonal IgG antibody (sc-101762) and goat anti-rabbit IgG-HRP antibody (sc-2004) were purchased from Santa Cruz Biotechnology (Heidelberg, Germany). The DNA repair enzyme formamidopyrimidine-DNA-glycosylase (FPG) from *Escherichia coli* was obtained from New England Biolabs (Frankfurt am Main, Germany). Sulfatase (from *Aerobacter aerogenes*, Type VI) and β-glucuronidase (from *E. coli*) were purchased from Sigma-Aldrich Chemie GmbH (Taufkirchen, Germany). All other chemicals and reagents were purchased from Carl Roth GmbH Co. KG (Karlsruhe, Germany) or Sigma-Aldrich Chemie GmbH unless stated otherwise.

### Cell culture

The human esophageal carcinoma cell line KYSE510 was obtained from the German Collection of Microorganisms and Cell Cultures GmbH (DSMZ, Braunschweig, Germany). Culture media and supplements were purchased from GIBCO Invitrogen (Karlsruhe, Germany). KYSE510 cells were cultivated in Rosswell Park Memorial Institute (RPMI) 1640 medium supplemented with 10 % fetal calf serum (FCS) and 1 % penicillin/streptomycin (50 units/mL and 50 µg/mL, respectively). The Chinese hamster ovary cell line CHO-ARE-luciferase as described in Heiss et al. (Heiss et al. 2014) was cultivated in Dulbecco’s Modified Eagle’s Medium (DMEM) with 10 % FCS, 4 µg/mL puromycin and 1 % l-glutamine. Both cell lines grew in humidified incubators at 37 °C and 5 % CO_2_. Cells were routinely tested for the absence of mycoplasma contamination.

### Cytotoxicity assay (WST-1)

Mitochondrial activity as a measure of cytotoxicity was determined in KYSE510 esophageal carcinoma cells with the WST-1 test kit (Roche Applied Science, Mannheim, Germany). 2-(4-Iodophenyl)-3-(4-nitrophenyl)-5-(2,4-disulfophenyl)-2H-tetrazolium is reduced to a water-soluble formazan salt by mitochondrial enzymes of the cells. The formazan salt can be detected photometrical. 1,300 KYSE510 cells/well were seeded in 96-well plates and cultivated for 48 h. Thereafter, cells were incubated with AOH and 4-OH-AOH or the positive control Triton X-100 for 24 h in serum-containing medium. The assay was performed according to the manufacturer’s protocol, and absorbance was measured at 450 nm with a reference wavelength of 650 nm with a plate reader (Victor^3^ V, Perkin Elmer, Waltham, MA, USA). Cell viability was specified as mitochondrial activity and calculated as treated cells to control cells × 100 (% T/C).

### Dichlorofluorescein (DCF) assay

The production of cellular ROS, indicative for increased oxidative stress, was measured by the DCF assay as reported in Keston and Brandt (1965). The assay is based on the uptake of the non-fluorescent dihydrodichlorofluorescein diacetate and intracellular de-esterification to dihydrodichlorofluorescein, which is oxidized to 2′,7′-dichlorofluorescein by reactive oxygen species. DCF can be measured fluorometrically at an excitation of 485 nm with the multimode plate reader Victor^3^ V (Perking Elmer, Waltham, MA, USA). KYSE510 cells were seeded 48 h prior to incubation in a black 96-well plate. Cells were incubated with AOH, AME, 4-OH-AOH, 4-OH-AME or menadione (MEN), used as positive control compound, in colorless serum-free medium for 1 h. Oxidative stress was determined as relative fluorescence and calculated as treated cells over control cells (oxidative stress (%) = 100 × (emission of treated sample)/(emission of control).

### Nrf2 reporter gene assay

The ARE-dependent luciferase assay was performed as described in Heiss et al. (2014). Transfected CHO cells were plated at a density of 6 × 10^5^ cells/well in 96-well plates and cultivated for 4 h in serum-containing medium. Subsequently, cells were incubated with AOH, AME, 4-OH-AOH, 4-OH-AME (solvent vehicle 0.5 % DMSO) or the positive control 2-cyano-3,12-dioxoolean-1,9-dien-28-oic acid (CDDO; 100 nM) for 20 h in serum-containing medium. After the incubation, the cells were washed with PBS twice and frozen at least for 1 h at −80 °C. Then 50 µL luciferase lysis buffer (Promega; E1531) was added to each well and shaken for 10 min. 40 µL of the cell lysate was transferred into a black 96-well plate. The fluorescence signal of GFP was measured at 485-nm excitation and 520-nm emission wavelength, and the ATP and luciferin solution were added and measured afterward automatically by the Genios Pro plate reader (Tecan, Grödig, Austria). The ratio of chemiluminescence/GFP fluorescence was formed and normalized to solvent control (Nrf2 activation = (ratio of treated sample)/(ratio of solvent control).

### Single cell gel electrophoresis (comet assay)

Single cell gel electrophoresis was performed according to Tice et al. (2000). A total of 300,000 KYSE510 cells were spread into Petri dishes (Ø 5.5 cm) and allowed to grow for 48 h. Subsequently, cells were treated with AOH, AME, 4-OH-AOH and 4-OH-AME for 1 h in serum-free medium. UV-B light (*λ* = 312 nm; dosage: 448 J/cm^2^) was used as a positive control. Subsequent working steps were done according to Pelka et al. (2009). Additional treatment was performed with the DNA repair enzyme FPG from *E. coli* (New England Biolabs, Vienna, Austria) according to the manufacturer’s protocol, using 2 × 0.08 units FPG for 30 min at 37 °C. This allows the detection of additional oxidative damage to DNA bases (Hatahet et al. 1994; Tchou et al. 1994). Fluorescence microscopy was performed with a Zeiss Axioskop 40 FL (*λ*_ex_ = 546 ± 12 nm; *λ*_em_ ≥ 590 nm) after staining with ethidium bromide. Slides were subjected to computer-aided image analysis (Comet Assay IV System, Perceptive Instruments, Suffolk, UK), scoring 2 × 50 randomly picked cells per slide. The results were parameterized with respect to intensity of DNA in the comet tail and calculated as percentage of overall DNA intensity in the respective cell.

### Decatenation assay

The cell-free decatenation assay was used to determine the catalytic activity of topoisomerase IIα to release DNA minicircles from kDNA. A mixture of topoisomerase IIα (TopoGEN) and supercoiled kDNA (200 ng) was incubated for 60 min at 37 °C with the respective AOH, AME, 4-OH-AOH and 4-OH-AME concentrations according to the manufacturer’s protocol with slight modifications (TopoGEN, Inc., Florida, USA). ETO (50 µM) served as a positive control. To stop the reaction, 5 µL loading buffer was added and samples were electrophoresed in 1 % agarose gel. The gel was dyed with ethidium bromide (10 µg/mL in distilled water) and documented by digital photography under UV light using the LAS 4000 (Fujifilm, Tokyo, Japan).

### In-vivo-complex-of-enzyme (ICE) assay

Briefly, 3 × 10^6^ KYSE510 cells were seeded in Petri dishes (Ø 15 cm) for 72 h. Afterward, cells were incubated with AOH, AME, 4-OH-AOH, 4-OH-AME or 50 µM of the topoisomerase poison ETO as a positive control for 1 h in serum-free medium. The cell lysate was layered on a cesium chloride gradient and centrifuged at 100,000×*g* for 22 h at 20 °C. 300 µL fractions of each sample were collected. The fractions were blotted on a nitrocellulose membrane, and the topoisomerase complexes were conjugated with topoisomerase IIβ-specific rabbit polyclonal antibodies (Santa Cruz Biotechnology). An anti-rabbit IgG peroxidase conjugate was used as secondary antibody. The respective chemiluminescent signals (Lumi-GLO, Cell Signaling Technology, Danvers, USA) were analyzed using the LAS 4000 (Fujifilm, Tokyo, Japan). Arbitrary light units were referred to the DNA content of the fractions and plotted as treated sample over control × 100 (%, T/C).

### Cellular uptake and metabolism of AOH, AME, 4-OH-AOH and 4-OH-AME (LC-MS/MS)

To analyze the cellular uptake of AOH, AME, 4-OH-AOH and 4-OH-AME, the concentration of the four substances in the medium and in the cells after 1 h of incubation was quantified by LC-ESI-MS/MS. KYSE510 cells (250,000) were seeded into Petri dishes (Ø 5.5 cm), and after 72 h the cells were treated with 10 µM AOH; AME, 4-OH-AOH, 4-OH-AME or 1 % DMSO for 1 h in serum-free medium. The incubation medium was transferred into a 15-mL tube and stored at −80 °C until further analysis. The cell layer was washed twice with PBS and frozen at −80 °C. After thawing of the cells, 400 µL bidest H_2_O was added to the cells which were scratched off the Petri dish using a cell scraper and transferred into a reaction tube. Cells were lysed by three freeze–thaw cycles (−80 °C, 1 h) and subsequent sonification (15 s). Cell lysates were centrifuged for 10 min at 18,000*g* at 4 °C. 10 µL of the supernatant was withdrawn to determine the protein amount by a Bradford assay. The supernatant and the cell pellet were homogenized by sonification again and extracted three times with 600 µL ethyl acetate. The fractions were collected for each sample and evaporated to dryness in the S-Concentrator SA-VC-300H with the diaphragm pump Pu-Hy-CH (H. Saur, Reutlingen, Germany). The residue was diluted in PBS, and three aliquots, a 100 µL of each cell lysate and medium supernatant was filled in reaction tubes. The aliquots were either incubated with glucuronidase, sulfatase or phosphate buffer for 2 h at 37 °C. The reaction was stopped by adding ethyl acetate (−20 °C). The samples were extracted, the residues dissolved in methanol and analyzed. LC-ESI-MS/MS analysis was carried out using a TSQ vantage triple quadrupole mass spectrometer interfaced with an Accela autosampler and an Accela 1250 pump all from Thermo Fisher Scientific (Germany). The system was operated utilizing the Xcalibur software 2.1 (Thermo Fisher Scientific, Germany). Separation was achieved on a Luna 3u C18 column (1.0 mm (i.d.) × 100 mm, Phenomenex, Torrance, CA) with the matching pre-column using gradient elution with H_2_O (solvent A) and acetonitrile (solvent B) as eluents, both containing 0.1 % formic acid. Gradient elution was performed as follows: 0 min, 30 % B; 5 min, 30 % B; 7 min, 100 % B; 12 min, 100 % B. The column oven temperature was set to 30 °C, and the injection volume was 20 μL. The flow rate was 0.1 mL/min. The following parameters were used to operate the triple quadrupole mass spectrometer: negative ion mode; multiple reaction monitoring; spray voltage, −3.0 kV; capillary temperature, 250 °C; vaporizer temperature, 100 °C; Q1 peak width [FWHM], 0.7; Q3 peak width [FWHM], 0.7; aux gas pressure, 5 arb; sheath gas pressure, 20 arb; collision gas pressure, 1 mTorr. The optimized collision energies and fragment ions for the investigated analytes are listed in Table 1.

**Table 1:** Optimized ESI-MS and ESI-MS/MS parameters, retention times and LOD/LOQ values of the applied LC-ESI-MS/MS method

Analyte	RT (min)	Precursor ion (*m/z*)	Ion species	Product ions^a^ (*m/z*)	CE^a,b^ (V)	LOD (ng/mL)^c^	LOQ (ng/mL)^d^
AOH	3.6	257	[M–H]^−^	213/147	23/33	2.1	6.8
AME	9.2	271	[M–H]^−^	256/228	23/33	1.4	4.8
4-OH-AOH	1.9	273	[M–H]^−^	258/214	25/31	1.9	6.3
4-OH-AME	5.8	287	[M–H]^−^	272/188	23/32	2.6	8.8

^a^Values are given in the order quantifier ion/qualifier ion

^b^Collision energy

^c^LOD was calculated based on a S/N ratio of 3:1

^d^LOQ was calculated based on a S/N ratio of 10:1

### Preparation of calibration solutions and spiking procedure

Solid reference standards were dissolved to a concentration of 1 mg/mL in methanol. From the 1 mg/mL standard, dilution series were created in methanol to yield concentrations of 1, 5, 10, 50, 100, 150, 200 ng/mL. These standards were utilized to generate a calibration curve for external calibration purpose. To evaluate the extraction efficiency and the matrix effects preliminary spiking experiments were performed. One concentration of each toxin (2.5 µM) was spiked to the scratched cells or the medium in triplicate before the extraction to cover the whole sample preparation procedure. Before analysis, the spiked samples were diluted 1:10. Quantitative data evaluation was performed using the Xcalibur Quan Browser software.

### Data analysis

For statistical evaluation, the Origin software was used. All results represent the mean of at least 3 independent experiments ± standard deviation (SD). Concentration-dependent data were statistically analyzed as stated in the figure legends.

## Results

### Impact of AOH and 4-OH-AOH on mitochondrial activity

**Fig. 2:**
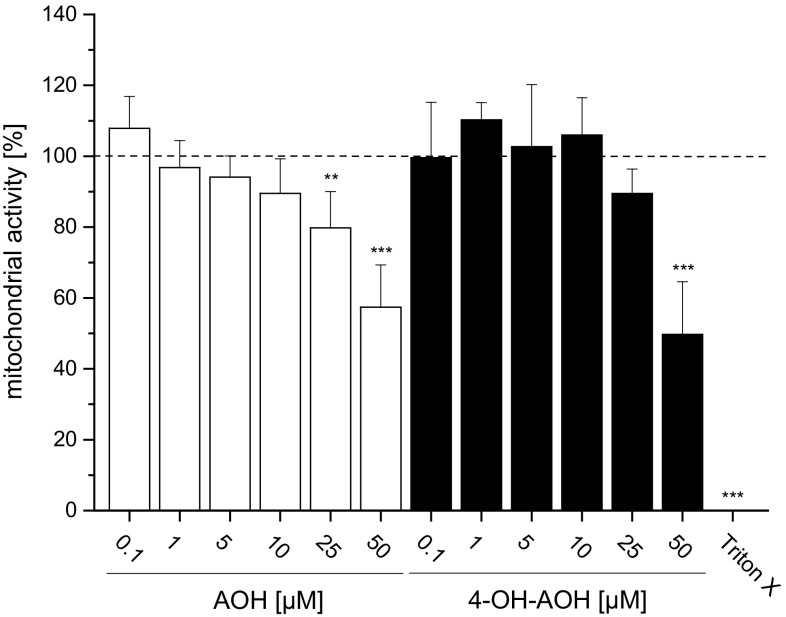
Impact of AOH and 4-OH-AOH on the viability of KYSE510 cells measured with the WST-1 assay in serum-containing medium after 24-h incubation. Triton X (0.1 %) was used as a positive control. The mitochondrial activity was calculated as incubated cells over control cells × 100 (%). The shown data are the mean ± SD of at least three independent experiments. Significances indicated display the significance level as compared to the respective control calculated by one-way ANOVA (*p < 0.05; **p < 0.01; ***p < 0.001)

Initial tests on the impact of hydroxylation on cytotoxicity were performed with the WST-1 assay, measuring mitochondrial activity. Cell viability of KYSE510 cells was affected by AOH and 4-OH-AOH after 24 h of incubation (Fig. 2). The mitochondrial activity was significantly reduced by 25 µM AOH by about 20 ± 10 %, whereas 25 µM 4-OH-AOH caused only a minor reduction in about 10 to 90 ± 7 % yet without statistical significance. At a concentration of 50 µM, cell viability dropped to about 57 ± 12 % for AOH and 50 ± 15 % for 4-OH-AOH, respectively. Triton X, a potent detergent, was used as a positive control and totally inhibited mitochondrial activity. In summary, no significant difference between metabolite and parent compound was evident regarding cytotoxic effects. Due to limited availability, 4-OH-AME could not be included in the testing.

### AOH, AME and 4-OH-AOH influence the intracellular redox status

**Fig. 3:**
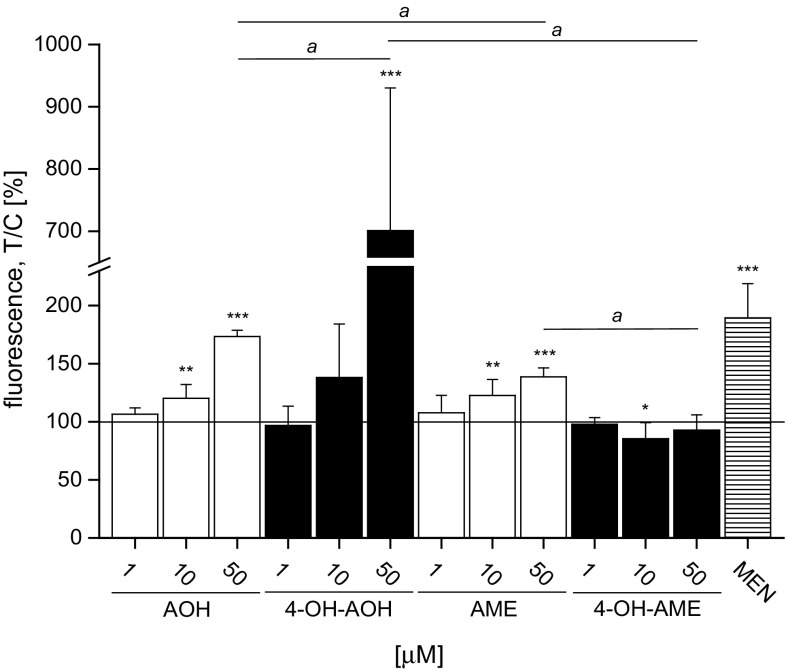
DCF assay with KYSE510 cells incubated for 1 h in serum-free colorless medium with AOH, AME, 4-OH-AOH, 4-OH-AME and the positive control menadione (MEN). The increase in fluorescence intensity was measured with a fluorimeter using 485-nm excitation and 535-nm emission filters. The level of formed reactive oxygen species was calculated as incubated cells over control cells (T/C). The data are presented as the mean ± SD of at least three independent experiments. Significances indicated display either (*asterisk*) the significance level as compared to the respective negative control calculated by one-way ANOVA (*p < 0.05; **p < 0.01; ***p < 0.001) or indicate significant differences (*a*) between the highest tested concentration (50 µM) of the four substances calculated by Student’s *t* test (*a* = *p* < 0.05)

Hydroxylation in 4-position of AOH or AME generates a catecholic structure which might undergo redox cycling. Thus, the potential impact of 4-hydroxylation on the intracellular ROS level was studied in KYSE510 cells using the dichlorofluorescein (DCF) assay. AOH, 4-OH-AOH and AME increased fluorescence intensity in a concentration-dependent manner (Fig. 3). The highest concentration tested (50 µM) raised the signal to about 174 ± 5 % for AOH and 701 ± 229 % for 4-OH-AOH in comparison with the solvent control (1 % DMSO). In contrast, the monomethylether AME had only marginal impact on the ROS level (139 ± 8 % at 50 µM) of KYSE510 cells. Incubation with 4-OH-AME did not even affect the fluorescence signal. The ROS level generated by 50 µM 4-OH-AOH was significantly higher than that of the parent compound, whereas the fluorescence signal caused by 4-OH-AME did not exceed the signal of its parent substance. MEN (20 µM), a known redox cycler, was used as a positive control and increased fluorescence intensity to nearly twofold of the base level.

### Activation of the ARE-dependent Nrf2 pathway by AOH, 4-OH-AOH and AME

**Fig. 4:**
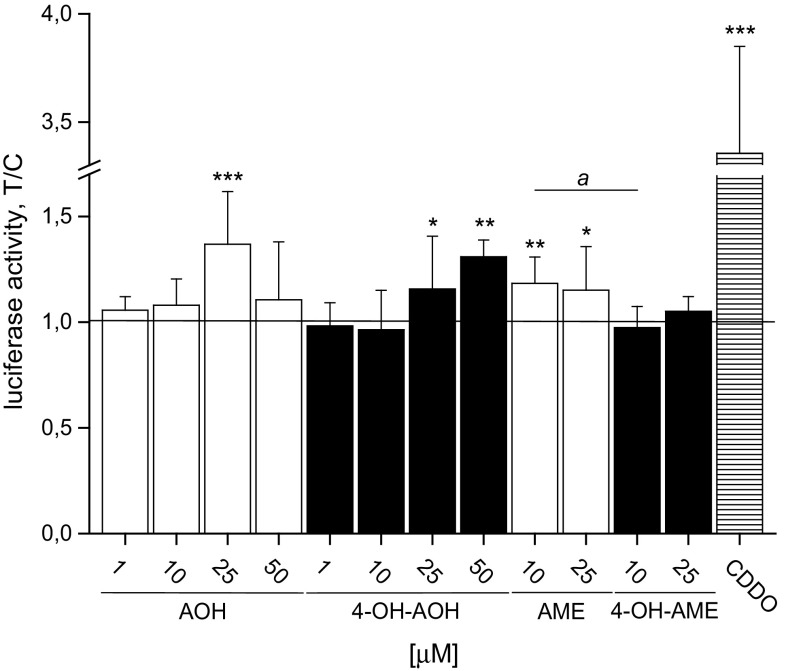
Activation of Nrf2 induced ARE-dependent luciferase in CHO cells CHO-ARE luciferase cells were treated with AOH, AME, 4-OH-AOH, 4-OH-AME (0.5 % DMSO) and the positive control 100 nM CDDO for 20 h. ARE-driven luciferase expression was measured, normalized to the GFP fluorescence and expressed as the fold induction of the negative control. Values are mean ± SD of at least three independent experiments. Significant differences from the solvent control 0.5 % DMSO were calculated using one-way ANOVA, followed by Fisher test (*p < 0.05; **p < 0.01; ***p < 0.001), and Student’s *t* test (*a* = *p* < 0.05) for the differences between the same concentrations of the four substances

To gain more precise information on the mechanisms involved in the modulation of the redox status of KYSE510 cells by the tested toxins, the impact on Nrf2, as a central regulator of cellular oxidative stress, was examined using an Nrf2 reporter gene assay. The Nrf2 pathway, leading to an activation of genes coding for phase II metabolizing enzymes, is induced by electrophiles or ROS. In transfected CHO-ARE-luciferase cells, the activated transcription factor Nrf2 specifically binds to the ARE coupled with the gene of *Photinus pyralis* luciferase in the nucleus and starts the expression of luciferase. After 20-h incubation, the bioluminescence signal was significantly raised concentration-dependently by 4-OH-AOH to 1.3 ± 0.08-fold at 50 µM (Fig. 4). The highest luciferase activity was detected at 25 µM AOH (1.4 ± 0.2-fold); nevertheless, no significant differences between the parent substance and the metabolite was apparent. AME incubation resulted in an increased luciferase activity starting at a concentration of 10 µM (1.2 ± 0.1-fold), whereas no effects were detected after 4-OH-AME incubation. AME and 4-OH-AME were not tested at 50 µM because of their limited solubility in 0.5 % DMSO, which was the limit for proper assay performance.

### Inhibition of topoisomerase IIα activity by AOH, AME, 4-OH-AOH and 4-OH-AME in a cell-free system

Previous studies demonstrated that AOH interferes with human topoisomerases, an effect which is likely to at least contribute to the genotoxic properties of the compound. The impact of 4-hydroxylation on the activity of human topoisomerase II was investigated in the decatenation assay under cell-free conditions, exemplified for the topoisomerase IIα isoform using etoposide (ETO, 50 µM) as a positive control. Active topoisomerase II releases minicircles from the complex high molecular kinetoplast DNA network (Fig. 6a, lane 10, 6b, lane 11), thus enabling the released minicircles to migrate in the agarose gel (Fig. 6a, b, lane 1). Inhibition of topoisomerase II activity was already apparent at a concentration of 10 µM 4-OH-AOH, and the enzyme activity was completely suppressed with 25 µM 4-OH-AOH (Fig. 6a, lane 8) as evident by the blunted release of free minicircles from the catenated kDNA by topoisomerase IIα. In contrast, AOH showed the first significant sign of suppression at 50 µM (lane 5), even though the line of free DNA minicircles vanishes already at 10 µM (lane 3). A significant difference between AOH and 4-OH-AOH was present at a concentration of 25 µM. Thus, under cell-free conditions, the inhibitory potential of the phase I metabolite 4-OH-AOH was more intense than for the parent compound. 10 µM AME inhibited the catalytic activity of topoisomerase IIα significantly (Fig. 6b, lane 3). A complete blockage of the enzyme was apparent at 25 µM 4-OH-AME (Fig. 6b, lane 8), but there was no statistically significant difference between the metabolite and AME. Taken together, under cell-free conditions, AOH appears as a less potent inhibitor of topoisomerase IIα compared to AME and both 4-OH-metabolites.

### Topoisomerase II poisoning by AOH, AME and the oxidative metabolites in KYSE510 cells

To verify topoisomerase inhibition on the cellular level, the ICE assay was used, determining the amount of cleavable complexes formed with DNA and topoisomerase IIα or IIβ in KYSE510 after 1-h incubation with 10 µM and 50 µM AOH, AME, 4-OH-AOH and 4-OH-AME. No topoisomerase IIα–DNA complexes were detected for any of the test substances (data not shown). Topoisomerase IIβ–DNA complexes were significantly raised to 277 ± 107 % by AOH, 208 ± 61 % by 4-OH-AOH, 203 ± 38 % by AME and 143 ± 36 % by 4-OH-AME with the highest concentration tested (Fig. 7). Yet, no statistically significant differences were found between the different test compounds.

### DNA-damaging potential of AOH, AME and their 4-hydroxy metabolites

Interference with topoisomerases as well as oxidative stress may cause DNA damage. The effects of AOH, AME and their 4-hydroxy metabolites on DNA integrity in KYSE510 cells were determined in the comet assay after 1-h incubation. Formamidopyrimidine-DNA-glycosylase (FPG) was included in the comet assay protocol to detect FPG-sensitive sites as an indication for oxidative DNA damage. A significant raise in tail intensities (5 ± 3.5 %, FPG-treated 6.6 ± 1.4 %) mediated by 50 µM AOH was detected, whereas 4-OH-AOH (50 µM; 3.1 ± 0.4 %, FPG-treated 3.7 ± 2.9 %) did not significantly affect DNA integrity. In contrast to AOH, neither significant increased DNA strand break levels nor additional oxidative DNA lesions after FPG treatment were observed for AME and 4-OH-AME.

### Cellular uptake of AOH, AME and the hydroxylated metabolites

Considering the substantial discrepancy between the effects of the hydroxylated metabolites on topoisomerase II under cell-free conditions and the impact on DNA integrity in the comet assay, the question was addressed whether differences in cellular uptake between the compounds might have to be considered. Cellular uptake of AOH, AME and their 4-hydroxylated metabolites was determined after 1-h incubation by analyzing the cell culture medium and the cell lysate by LC-MS/MS. A typical LC-MS/MS chromatogram is illustrated in Online Resource 2 (Supplement Fig. S1). In the cell lysate, the average amount of AOH was 304 ± 127 ng/mg protein and for AME 885 ± 269 ng/mg protein (Fig. 8a). The concentration of 4-OH-AOH (2 ± 1 ng/mg protein) and 4-OH-AME (13 ± 1 ng/mg protein) in the cell lysate was substantially lower compared to the parent substances. The apparent recovery in the cell lysate matrix was 78 ± 1 % for AOH, 60 ± 7 % for AME as well as 59 ± 10 and 73 ± 10 % for 4-OH-AOH and 4-OH-AME, respectively. No significant difference could be observed in the cell lysates after glucuronidase and sulfatase treatment. Neither newly formed hydroxylated products of AOH or AME nor a degeneration of 4-OH-AOH and 4-OH-AME to AOH and AME, respectively, were found in the medium or the cell lysate after 1-h incubation time. Instead, incubations with 4-OH-AOH showed an additional peak in the chromatogram eluting after 3.6 min (see Online Resource 3; Supplement Fig. S2). A compound with an *m/z* of 287 and the same SRMs as 4-OH-AME was observed.

In the cell culture medium 5 ± 2.5 µM AOH (apparent recovery 95 ± 28 %), 1.9 ± 0.5 µM AME (apparent recovery 104 ± 20 %), 1.3 ± 0.2 µM 4-OH-AOH (apparent recovery 44 ± 12 %) and 0.4 µM 4-OH-AME (apparent recovery 63 ± 18 %) were detected after 1-h incubation (Fig. 8b). Moreover, a significant difference in the analyzed culture medium of the 4-OH-AOH incubations was detected after sulfatase treatment when compared to the standard phosphate buffer samples. Also an increase in 4-OH-AOH in the medium after glucuronidase incubation was apparent but not yet significant. The other three substances revealed no differences between the standard incubation and the glucuronidase and sulfatase treatment (Fig. 8b).

## Discussion

*Alternaria* toxins belong to the group of so-called emerging mycotoxins, being not regulated so far, but with more and more data demonstrating their frequency of occurrence in feed and food and their genotoxic properties. Previous studies already indicated under in vitro conditions the formation of oxidative metabolites of AOH and AME and the induction of oxidative stress (Burkhardt et al. 2011; Pahlke et al. 2015; Pfeiffer et al. 2007b). The present study investigated and compared the effects of AOH and AME to the phase I metabolites 4-OH-AOH and 4-OH-AME in human esophageal cancer cells with respect to the induction of oxidative stress, the ability to target topoisomerase II as a central genotoxic mechanism of AOH and the consequences for DNA integrity under consideration of cellular uptake and metabolism.

The fluorescence signal in the DCF assay, indicative for enhanced intracellular ROS levels in KYSE510 cells mediated by AOH (Fig. 3), was in accordance with previous data on the induction of oxidative stress by AOH in different cell lines (Fernández-Blanco et al. 2014; Pahlke et al. 2015; Solhaug et al. 2012; Tiessen et al. 2013a). In KYSE510 cells, AME was significantly less potent than AOH to enhance the intracellular ROS level, indicating that the methyl-moiety at C9-position might play a role. Of note, the cellular uptake of AME in KYSE510 cells was almost two times higher than for AOH (Fig. 8a), potentially due to the higher lipophilicity of AME. These data are contrary to the findings of Burkhardt et al. (2009) who reported a faster uptake for AOH than for AME in human colorectal adenocarcinoma cells (Caco2) after 1-h incubation and a concentration of 20 µM. These results argue for the fact that the toxicokinetics of AOH and AME are cell-type specific. The phase I metabolite 4-OH-AME was found in a sixfold higher concentration in KYSE510 cells than 4-OH-AOH; however, both in a much lower concentration when compared to the parent compounds. Of note, the amount of ROS was increased fourfold by 50 µM 4-OH-AOH as compared to AOH. This is in line with the assumption that catechols might lead to a higher generation of ROS due to redox cycling. Consequently, it would be reasonable to expect 4-OH-AME to produce higher amounts of ROS than AME. However, in the DCF assay, no increase in the fluorescence signal was detected (Fig. 3).

The results of the DCF assay were supported by the data of the Nrf2 reporter gene assay (Fig. 4). Enhanced intracellular levels of ROS are assumed to activate the redox-sensitive transcription factor Nrf2 and subsequent reporter gene expression. Luciferase activity was indeed significantly increased by AOH, AME and 4-OH-AOH, whereas 4-OH-AME turned out to be ineffective. The increase in luciferase activity in the CHO reporter gene system observed for AOH, its hydroxylated metabolite and AME did not completely reflect the DCF data obtained with KYSE510 cells, whereby the different cell types and assay protocols have to be taken into account. The observed Nrf2/ARE-activating potency of AOH in the CHO reporter gene assay is in line with earlier reports on the induction of the Nrf2-pathway in HT29 colon carcinoma cells resulting in enhanced levels of Nrf2/ARE-regulated detoxifying enzymes such as GST and γ-GCL (Tiessen et al. 2013a).

**Fig. 5:**
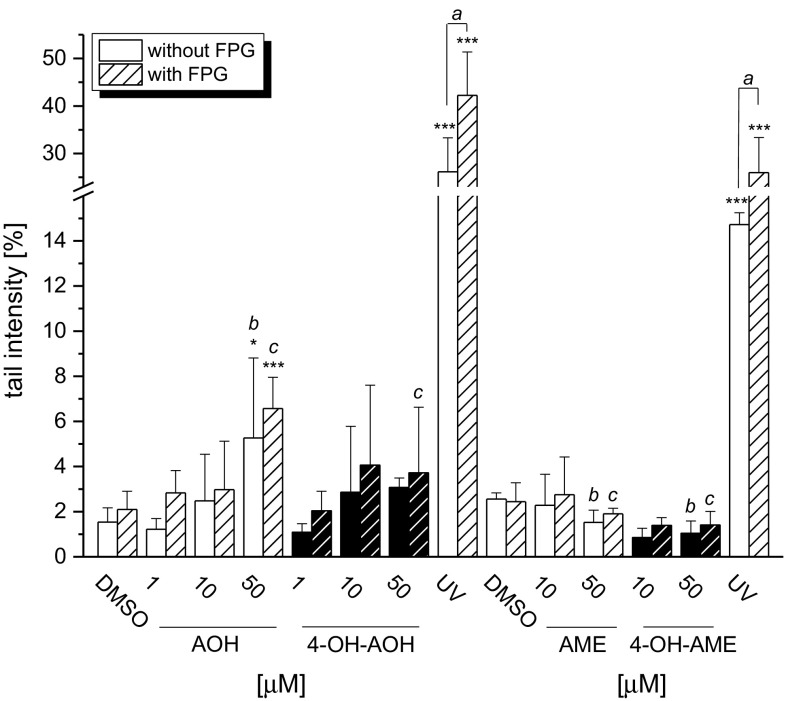
Mediated DNA strand breaks measured with the comet assay in KYSE510 cells after 1-h incubation with AOH, AME and the metabolites 4-OH-AOH and 4-OH-AME in serum-free medium. 1 % DMSO was used as a solvent control and UV light as a positive control. Further treatment with FPG allows detection of additional oxidative damage to DNA bases (*dashed bars*). Values are the means of at least three independent experiments ± SD, each performed in duplicate. Significances indicated refer to either (*asterisk*) the significance level compared to the respective negative control calculated by one-way ANOVA, followed by Fisher test (*p < 0.05; **p < 0.01; ***p < 0.001), display significant differences (*a*) between FPG-treated and untreated samples calculated by Student’s *t* test (*a* = *p* < 0.05) or show significant differences between 50 µM AOH without FPG (*b*) 50 µM AOH with FPG treatment (*c*) and the three other substances calculated by one-way ANOVA, followed by Fisher test (*b*, *c* = *p* < 0.05)

To further clarify the role of pro-oxidant properties of the mycotoxins and their hydroxylated metabolites with regard to genotoxicity, the DNA strand-breaking properties were analyzed in the comet assay (Fig. 5). However, the pro-oxidant properties of AOH, 4-OH-AOH and, to a lesser extent, AME, were not reflected by increased FPG-sensitive sites in the DNA of KYSE510 cells. Previous studies indicated that oxidative stress does not play a predominant role in the induction of DNA damage by AOH and AME in HT29 cells (Tiessen et al. 2013a). However, enhanced levels of FPG-sensitive sites were reported in the murine macrophage cell line RAW 264.7 after 2-h exposure to AOH (Solhaug et al. 2012), indicating potential species differences with respect to oxidative DNA damage. It is also likely that ROS generation could affect the cell membrane integrity by lipid peroxidation already stated by Fernández-Blanco et al. (2014), who investigated in Caco2 colon carcinoma cells the effects of AOH on lipid peroxidation and antioxidant capacity of CAT and SOD. They found high levels of malondialdehyde, a biomarker for lipid peroxidation (LPO), and increased SOD activity catalyzing the dismutation of superoxide anions to hydrogen peroxide. In conclusion, ROS production after exposure to AOH was correlated with the enhancement of LPO in Caco2 cells and therefore might play a role in cell membrane damage instead of direct oxidative DNA damage. This could further lead to an increased permeability for toxins resulting in cell death at a later stage (Matés 2000). After 24-h incubation of KYSE510 cells with 50 µM AOH or 4-OH-AOH, a significant decrease in about 40 and 50 %, respectively, in mitochondrial activity was detected in the WST-1 assay (Fig. 2). Although 4-OH-AOH induced a higher ROS production than its parent compound, no significant difference was obvious between both regarding the impact on cell viability of KYSE510 cells exposed to the highest concentration tested.

**Fig. 6:**
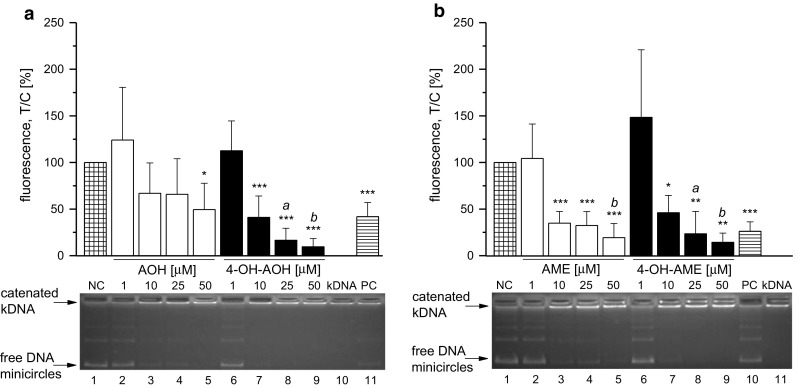
Catalytic activity of recombinant human topoisomerase IIα determined as the decatenation of kDNA. Topoisomerase IIα was incubated for 60 min at 37 °C with (**a**) AOH, 4-OH-AOH, (b) AME, 4-OH-AME or the topoisomerase poison etoposide (PC, 50 µM). The reaction was stopped with a loading dye, and samples were directly separated by 1 % agarose gel electrophoresis. The stained gel was documented under UV light and fluorescence signals of decatenated kDNA treated with topoisomerase IIα, and the substances were calculated as T/C (%) in comparison with the solvent control DMSO (NC). The data show the mean ± SD of at least three independent experiments. Significances compared to the respective control (*asterisk*) were calculated by one-way ANOVA, followed by Fisher test (*p < 0.05; **p < 0.01; ***p < 0.001). Additional significant differences between the four substances with the same concentration were calculated by one-way ANOVA. Only the results of 25 µM AOH (*a*) or 50 µM AOH (*b*) differ from the other substances with the same concentration (*a*, *b* = *p* < 0.05). A representative agarose gel is depicted under the graph. Lane 1 showed the result of the solvent control DMSO with kDNA exposed to topoisomerase II. Active topoisomerase II releases free DNA minicircles from catenated kDNA. Increasing concentrations of the parent compound (lane 2–5) and the metabolite (lane 6–9) demonstrate an inhibitory effect of the catalytic activity as well as the positive control ETO (**a** line 11 and **b** line 10). Lanes 10 (**a**) and 11 (**b**) represent catenated kDNA not incubated with topoisomerase IIα

**Fig. 7:**
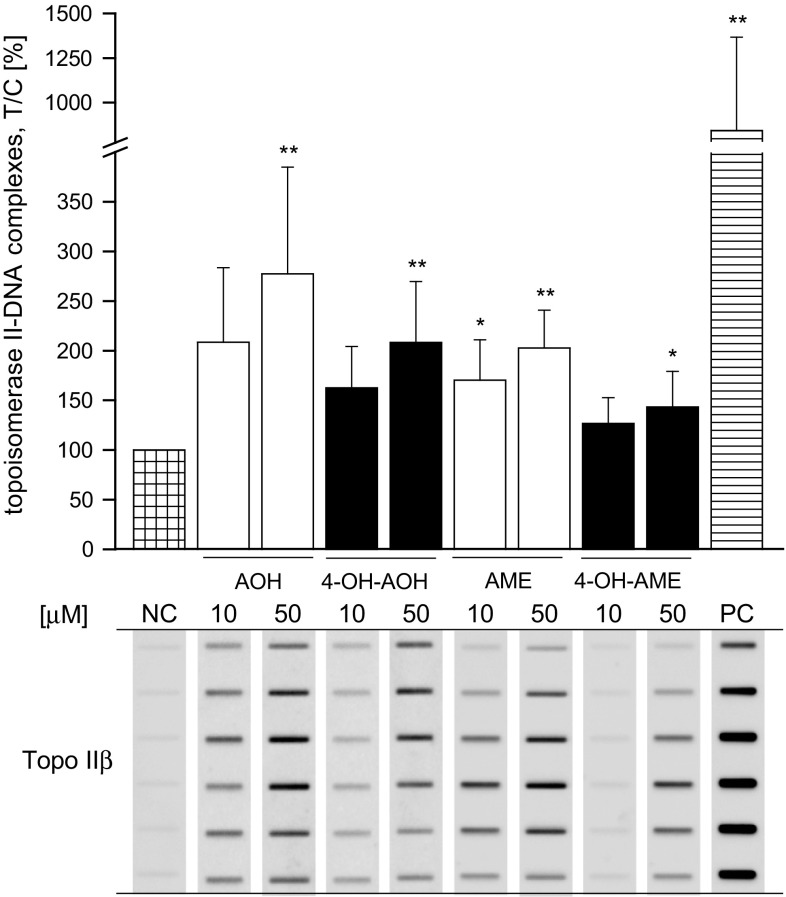
Detection of the covalent topoisomerase II–DNA complexes stabilized by the parent substances AOH, AME and their phase I metabolites in KYSE510 cells. Cells were treated with the test compounds or the positive control etoposide (PC) for 1 h in serum-free medium. Cell lysate fractions were collected after centrifugation and blotted on a nitrocellulose membrane. Representative immunoblots of the DNA–positive fractions are depicted below the graph. The level of topoisomerase IIβ–DNA complexes was calculated as treated cells over control cells with respect to the DNA content × 100 (T/C, %). The data presented are the mean ± SD of at least three independent experiments. Significances indicated refer to the negative control DMSO (NC) and were calculated using one-way ANOVA followed by Fisher test (*p < 0.05; **p < 0.01; ***p < 0.001)

**Fig. 8:**
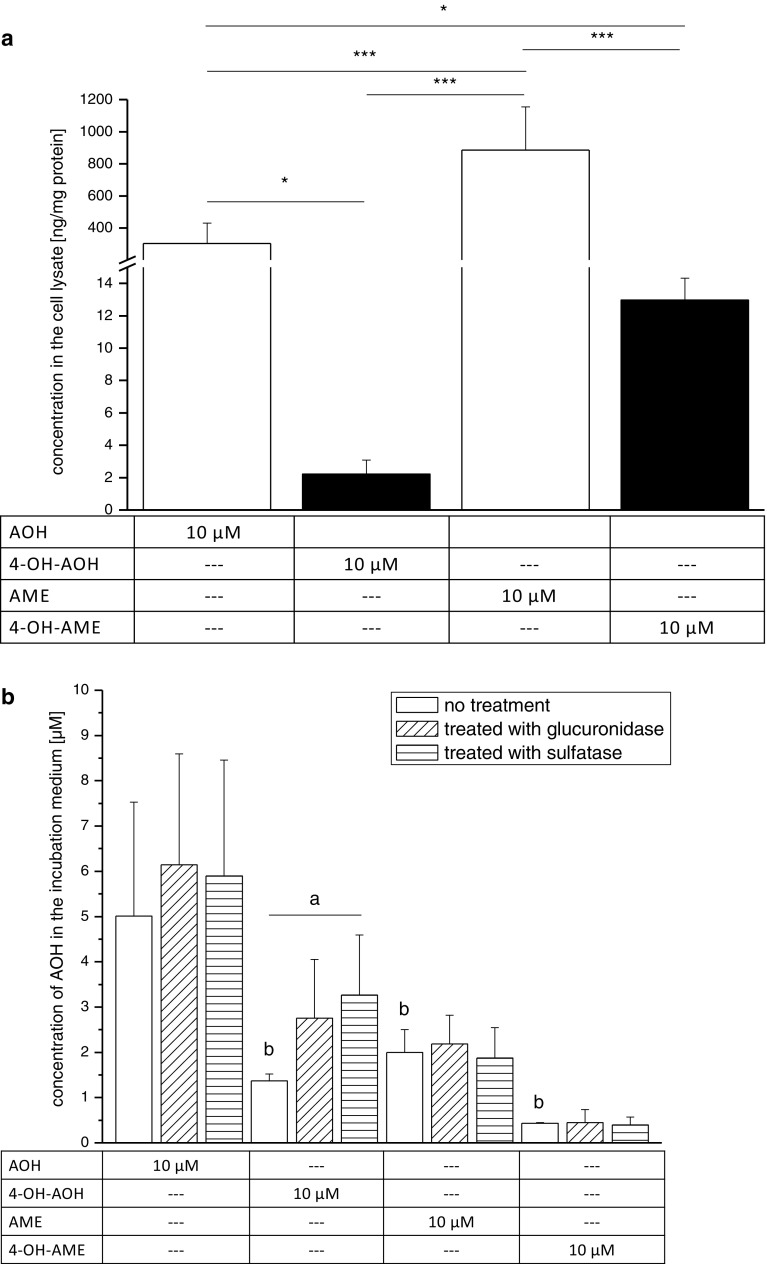
Quantification of the parent compounds AOH and AME and their metabolites in the cell lysate (**a**) and the medium (**b**) after 1-h incubation in KYSE510 cells. Cells were treated with the respective test compounds (10 µM) for 1 h at 37 °C, incubation media were collected, and cells were harvested and extracted with ethyl acetate. Cell lysates and incubation media were analyzed with HPLC-MS. Values shown are the mean ± SD of at least three independent experiments. Normality was tested according to Shapiro–Wilk (*p* > 0.05). Significances indicated refer either to the differences between substance incubations calculated by one-way ANOVA, followed by Fisher test (*p < 0.05; **p < 0.01; ***p < 0.001), differences between untreated and with enzyme (glucuronidase, sulfatase) treated samples (repeated measurements ANOVA, followed by Fisher test; *a* = *p* < 0.05), differences to the parent substance AOH (*b* = *p* < 0.05)

Previous studies demonstrated the topoisomerase poisoning properties of AOH, which is expected to at least contribute to its DNA-damaging properties. Under cell-free conditions 4-OH-AOH had a greater impact on topoisomerase II activity than AOH (Fig. 6a), whereas AME and 4-OH-AME seemed to be working quite equal in the assay (Fig. 6b). However, in the ICE assay, detecting the impact of the test compounds on topoisomerase II within cells, it became apparent that the stronger topoisomerase inhibitory effects of 4-OH-AOH and 4-OH-AME are limited to cell-free conditions. It is conceivable that the effects on cellular topoisomerase II could be attenuated by additional cellular responses, leading to detoxification of the substances or induction of DNA repair mechanisms as already shown in different cell lines (Fehr et al. 2010; Tiessen et al. 2013a; Solhaug et al. 2012). We previously demonstrated that AOH and AME act as topoisomerase II poisons in HT29 and A431 cells (Fehr et al. 2009), contributing to the genotoxic properties by affecting DNA integrity (Tiessen et al. 2013b). In A431 cells, a preference of AOH for the topoisomerase IIα isoenzyme was indicated but only observed in a limited concentration range (Fehr et al. 2009). In KYSE510 cells, clearly enhanced levels of the covalent topoisomerase–DNA intermediate were only detected for the topoisomerase IIβ isoform (Fig. 7), which might hint at cell-type specific differences. Both isoforms have distinct patterns of expression and separate functions. Topoisomerase IIα is only expressed in proliferating cells and reaches a maximum during G_2_/M phase in the cell cycle. In contrast, the expression of IIβ is independent of the cell cycle and present in all human tissues (Austin and Marsh 1998; Ketron and Osheroff 2014). There was a tendency of both 4-OH-metabolites to enhance the level of covalent topoisomerase IIβ–DNA complexes, but to a lower extent compared to the respective parent compound and without reaching statistical significance. AME, 4-OH-AOH and 4-OH-AME appeared to be less effective to trap topoisomerase IIβ–DNA complexes compared to AOH, which is in line with the absence of DNA strand breaks in the comet assay for these three compounds (Fig. 5). Genotoxic effects of AOH and AME have already been described by Fehr et al. (2009) in HT29 and A431 cells in the same concentration range where topoisomerase poisoning occurred. The question arises which mechanisms prevent the formation of DNA strand breaks in KYSE510 after AME, 4-OH-AOH and 4-OH-AME incubation, although inducing the stabilization of topoisomerase IIβ–DNA complexes. Pfeiffer et al. (2007a) described DNA strand-breaking activities for AOH and AME in the alkaline unwinding assay after 1 h for both HT29 and HepG2, but after 24 h, DNA damage was only present in HepG2. The various outcomes of both cell lines can be explained by (1) the glucuronidation behavior and the associated detoxification of the toxins and (2) the onset of DNA repair pathways. After 24 h, no unconjugated substance was detected in the media of HT29 cells, whereas in HepG2 approximately 75 % were still present as AOH and AME (Pfeiffer et al. 2007a). In the esophagus, UGT1A7 is the most abundant UGT isoform; however, the expression level is relatively low compared to human liver, small intestine and colon cells (Ohno and Nakajin 2009). Furthermore, AOH is a twice better substrate for UGT1A7 compared to AME (Pfeiffer et al. 2009). Therefore, enhanced glucuronidation is not a suitable explanation for the lack of DNA damage for AME or 4-OH-AME incubation. Accordingly, no sulfation or glucuronidation conjugates of AME and 4-OH-AME were detected after 1-h incubation (Fig. 8). Moreover, the amount of 4-OH-AME in the cell lysate was quite low compared to the concentration of AME, which might also explain the low impact on DNA damage.

Previously reported by Fehr et al. (2010), the human repair enzyme tyrosyl-DNA phosphodiesterase 1 (TDP1) is a vital factor for the modulation of AOH-mediated DNA damage by reducing covalent topoisomerase–DNA adducts. The study underlines the importance of TDP1 on the repair of topoisomerase-mediated DNA damage which might reduce the stabilized cleavable complexes by AME, 4-OH-AOH and 4-OH-AME. However, another DNA repair process to connect DNA double strands after breakage is non-homologous end joining (NHEJ). The involvement of NHEJ after prolonged incubation in HCT116 cells was examined by Tiessen et al. (2013b). HCT116 cells treated with siRNA to suppress PCNA and Ku70, two proteins involved in the repair process of DNA double strand breaks via NHEJ, were used to determine AOH-mediated DNA lesions. The suppression of Ku70 and PCNA enhanced the DNA-damaging effect of AOH in the comet assay suggesting that this process must be involved in DNA repair. In summary, the lower efficiency of AME, 4-OH-AME and 4-OH-AOH to stabilize topoisomerase II–DNA complexes in association with potential DNA repair processes (NHEJ) and enzymes like TDP1 are feasible explanations for the low or rather non-DNA damage induced by these compounds in KYSE510 cells.

Finally, the role of toxicokinetics and metabolism of these substances in KYSE510 cells was considered. As cytochrome P450 enzymes should occur commonly in esophageal cells (Lechevrel et al. 1999) and Pahlke et al. (2015) reported after 24-h incubation an induction of *CYP1A1* transcripts and activity in KYSE510 cells by AOH and (less pronounced) by AME, hydroxylated products of AOH and AME should occur within the cells. However, hydroxylated compounds were neither present in the cell lysate nor in the incubation medium after 1-h exposure, demonstrating that at least the mediated DNA damage by AOH after 1 h can be ascribed to the parent compound. Additionally, glucuronidation and sulfation products of AOH and AME were not detected (Fig. 8) likely due to the short incubation time and low levels of UGTs in esophageal cells (Ohno and Nakajin 2009). After incubation of KYSE510 cells with 4-OH-AOH or 4-OH-AME, the compounds were found in the cell lysate without evidence on the regeneration to AOH or AME in the cell culture medium and cell lysate. The low concentration of 4-OH-AOH and 4-OH-AME in the cell lysate after incubation might be indicative for a poor cellular uptake resulting in the minor effects observed in the ICE- and comet assay. However, only low amounts of the initial concentration (10 µM) of 4-OH-AOH and 4-OH-AME were present in the medium after 1 h, which could be the outcome of further metabolism like glucuronidation, sulfation, GSH-conjugation or methylation. Following this consideration, incubations with 4-OH-AOH exhibited increased amounts of sulfation conjugates in the incubation medium (Fig. 8b) and an additional metabolism product (Supplement Fig. S2). Based on its *m/z* ratio of 287 and the retention behavior, we speculate that this peak might correspond to a methylated product of 4-OH-AOH generated by catechol-*O*-methyl transferase (COMT). Indicative for the formation of methylated products is the fact that (1) Burkhardt et al. (2011) described the formation of 4-OH-AOH and 4-OH-AME methylation products at the catechol structure after incubation with rat liver cytosol containing COMT, and (2) COMT has been detected in all human tissues with the highest activity in the liver, followed by kidneys and gastrointestinal tract (Männistö and Kaakkola 1999). Therefore, it seems likely that methylation of 4-OH-AOH is a way of detoxification in KYSE510 cells, which might explain the minor effects in the other assays as compared to AOH. However, it could not be fully excluded that the detected compound belongs to another hydroxyl isomer of AME such as 2-OH-AME. Thus, further studies are needed to confirm the structure of the newly detected metabolite.

In summary, the potential phase I metabolites 4-OH-AOH and 4-OH-AME were synthesized and systematically compared to the parent compounds with respect to oxidative stress, DNA damage, topoisomerase inhibition, cellular uptake and metabolism. The results reveal that in KYSE510 cells the catecholic structure of 4-OH-AOH and 4-OH-AME does not lead to enhanced levels of oxidative DNA damage although the redox cycling activity of 4-OH-AOH is at hand. Despite the huge induction of ROS by 4-OH-AOH, the transcriptional activity of Nrf2 was not affected accordingly. The effects of the metabolites were minor compared to the respective parent substances AOH or AME in terms of topoisomerase inhibition and DNA strand-breaking effects.

## Electronic supplementary material

Below is the link to the electronic supplementary material.

Supplementary material 1 (DOCX 179 kb)
