# Supplementary material for: Impact of phase I metabolism on uptake, oxidative stress and genotoxicity of the emerging mycotoxin alternariol and its monomethyl ether in esophageal cells
Source: Arch Toxicol. 2016 Jul 15;91(3):1213–26. doi: 10.1007/s00204-016-1801-0 (PMC5316404; doi:10.1007/s00204-016-1801-0)
Supplement: Supplementary file 1 — Supplementary material 1 (DOCX 179 kb) [file 204_2016_1801_MOESM1_ESM.docx]

**Supplement**

**Online Resource 1**

**Chemical Synthesis**

*General remarks*

All reactions were performed under an argon atmosphere. The progress of reactions was monitored by thin-layer chromatography (TLC) over silica gel 60 F254 (Merck, Germany). The chromatograms were visualized by irradiation with ultraviolet light or by heat staining with ceric ammonium molybdate in ethanol/sulfuric acid. LC-ESI-MS/MS was performed on an HCT ion trap mass spectrometer (Bruker, Germany) in full scan mode. Chromatographic separation was done on a 1200 series HPLC system (Agilent Technologies, Germany) using a Luna RP-C18 column (3.0 x 150 mm, 3 μm particle size, Phenomenex, Germany) and application of pure substances was achieved using a TLC-MS interface (Camag, Germany). Preparative column chromatography was performed on silica gel 60 (Merck, 40-63 μm) or RP-C18 silica gel (Merck, 40-63 μm) using a Büchi SepacoreTM Flash System. NMR spectra were recorded on a Bruker DPX-200 MHz or Avance DRX-400 MHz spectrometer. Data were recorded and evaluated using TOPSPIN 1.3 (Bruker Biospin). All chemical shifts are given in ppm relative to tetramethylsilane. The calibration was done using residual solvent signals. Multiplicities are abbreviated as s (singlet), d (doublet), t (triplet), q (quartet), b (broad signal). A Thermo Scientific LTQ Orbitrap XL hybrid FTMS (Fourier Transform Mass Spectrometer) equipped with Thermo Scientific MALDI Interface or Thermo Fischer Exactive Plus Orbitrap (LC-ESI+) was used for high-resolution mass spectrometry.

*1,2,3-Trimethoxy-5-methylbenzene (****2****)*

A suspension of 3,4,5-trimethoxybenzaldehyde (**1**) (23.54 g, 120 mmol), Pd/C (10% Pd, 1.22 g, 1.14 mmol) and conc. HCl (2.00 g, 19 mmol) in MeOH (400 mL) was hydrogenated at room temperature and atmospheric pressure overnight. Additional Pd/C (10% Pd, 0.5 g) was added and stirring was continued overnight. The reaction mixture was filtered over celite. The solvent was evaporated to afford **2** as pale yellow oil (21.49 g, 98%). The spectroscopically pure product was used in the next step without any further purification. ^1^H NMR (200 MHz, CDCl_3_): δ = 2.31 (s, 3H), 3.83 (s, 3H), 3.84 (s, 6H), 6.39 (s, 2H) ppm; ^13^C NMR (50 MHz, CDCl_3_): δ = 21.9 (q, 1C), 56.0 (q, 2C), 60.9 (q, 1C), 105.9 (d, 2C), 133.6 (s, 1C), 135.8 (s, 1C), 153.1 (s, 2C) ppm.

*5-Methyl-1,2,3-benzenetriol (****3****)*

A mixture of **2** (12.12 g, 66.5 mmol), aqueous HI (57%, 57 mL) and aqueous H_3_PO_2_ (50%, 2 mL) was heated in a reaction apparatus equipped with an 18 cm-Vigreux column and iodomethane was continuously removed by distillation. The mixture was diluted with water (200 mL) and extracted with Et_2_O. The combined organic layer was washed with saturated aqueous NaHCO_3_ solution, dried and concentrated to obtain **3** as slightly pink colored solid (7.96 g, 85%). The spectroscopically pure product was used in the next step without any further purification. ^1^H NMR (200 MHz, DMSO-d_6_): δ = 2.05 (s, 3H), 6.06 (s, 2H), 7.76 (s, 1H), 8.61 (s, 2H); ^13^C NMR (50 MHz, DMSO-d_6_): δ = 20.7 (q, 1C), 107.7 (d, 2C), 127.3 (s, 1C), 130.5 (s, 1C), 146.0 (s, 2C).

*2-Ethoxy-6-methyl-1,3-benzodioxol-4-ol (****4****)*

A mixture of **3** (6.31 g, 45 mmol), HC(OEt)_3_ (10.00 g, 67.5 mmol), Amberlite IR 120-plus (H^+^) (0.316 g), molecular sieve powder (3 Å, 2.52 g) in toluene (170 mL) was heated in a reaction apparatus equipped with a 18 cm Vigreux column and the EtOH/toluene azeotrope was continuously removed by distillation. The mixture was filtered, applied to silica (42 g) and purified by column chromatography (90 g silica, hexanes/EtOAc = 10:1 to 5:1) to yield **4** as red oil (6.44 g, 73%). ^1^H NMR (200 MHz, CDCl_3_): δ = 1.25 (t, J=7.1 Hz, 3H), 2.23 (s, 3H), 3.73 (q, J=7.1 Hz, 2H), 5.37 (br, 1H), 6.33 (br, 2H), 6.85 (s, 1H); ^13^C NMR (50 MHz, CDCl_3_): δ = 14.9 (q, 1C), 21.4 (q, 1C), 59.5 (t, 1C), 102.3 (d, 1C), 108.9 (d, 1C), 111.2 (d, 1C), 130.6 (s, 1C), 132.4 (s, 1C), 138.2 (s, 1C) 147.0 (s, 1C).

*1-Iodo-3,5-dimethoxybenzene (****6****)*

To a solution of 1-bromo-3,5-dimethoxybenzene (**5**) (23.88 g, 110 mmol) in anhydrous THF (160 mL) *t*-BuLi (1.7 M in pentane, 145.6 mL, 247.5 mmol) was added slowly at -80 °C (acetone/N_2_ cooling bath). After 2 h a solution of iodine (83.76 g, 330 mmol) in anhydrous THF (170 mL) was slowly added and the mixture was stirred at room temperature overnight, then quenched with water (200 mL) and extracted with dichloromethane. The combined organic layer was washed with saturated aqueous sodium thiosulfate solution, dried and concentrated under reduced pressure. Recrystallization from ethanol afforded **6** as white solid (21.07 g, 73%). ^1^H NMR (200 MHz, CDCl_3_): δ = 3.76 (s, 6H), 6.40 (t, J=2.2 Hz, 1H), 6.86 (d, J= 2.3 Hz, 2H); ^13^C NMR (50 MHz, CDCl_3_): δ = 55.6 (q, 2C), 94.2 (s, 1C), 100.8 (d, 1C), 115.9 (d, 2C), 161.2 (s, 2C).

*2-Iodo-4,6-dimethoxybenzaldehyde (****7****)*

Compound **6** (20.98 g, 79.5 mmol) was suspended in anhydrous DMF (75 mL) and POCl_3_ (42.64 g, 278.1 mmol) was added at 0 °C. After complete addition the cooling bath was removed and the mixture was heated to 85 °C. Full conversion was observed after 5 h (TLC). The reaction mixture was quenched by addition of ice water and extracted with EtOAc. The combined organic layer was dried and concentrated under reduced pressure. Recrystallization from hexanes/EtOAc (3:1) afforded **7** as a yellow solid (16.10 g, 69%). ^1^H NMR (200 MHz, CDCl_3_): δ = 3.85 (s, 3H), 3.88 (s, 3H), 6.47 (d, J=2.2 Hz, 1H), 7.11 (d, J= 2.3 Hz, 1H), 10.12 (s, 1H); ^13^C NMR (50 MHz, CDCl_3_): δ = 55.9 (q, 1C), 56.0 (q, 1C), 98.6 (s, 1C), 99.1 (d, 1C), 118.3 (s, 1C), 119.2 (d, 1C), 163.3 (s, 1C), 164.6 (s, 1C), 190.6 (d, 1C).

*2-Iodo-4,6-dimethoxybenzoic acid (****8****)*

To a solution of **7** (2.92 g, 10 mmol) in *t*-BuOH (150 mL) and acetonitrile (30 mL), NaH_2_PO_4_·H_2_O (11.0 g, 80 mmol) and 2-methyl-2-butene (25 mL) were added. After cooling to 0 °C a solution of NaClO_2_ (80 mL, 1M aqueous solution) was slowly added. The mixture was stirred at room temperature for 8 h. The solvent was evaporated under reduced pressure and the residue was partitioned between EtOAc and water, followed by extraction of the aqueous layer with EtOAc. The combined organic layer was dried and concentrated under reduced pressure, yielding **8** as a spectroscopically pure product, which was used in the next step without any further purification. ^1^H NMR (200 MHz, DMSO-d_6_): δ = 3.74 (s, 3H), 3.78 (s, 1H), 6.64 (d, 1 H), 6.95 (d, 1H); ^13^C NMR (50 MHz, acetone-d_6_): δ = 55.7 (q, 1C), 56.0 (q, 1C), 93.0 (s, 1C), 98.9 (d, 1C), 114.8 (d, 1C), 124.5 (s, 1C), 156.9 (s, 1C), 160.8 (s, 1C), 168.3 (s, 1C).

*2-Ethoxy-6-methyl-1,3-benzodioxol-4-yl 2-iodo-4,6-dimethoxybenzoate (****9****)*

Compound **8** (2.67 g, 8.7 mmol) was dissolved in anhydrous dichloromethane (100 mL) and DMF (0.5 mL) and cooled to 0 °C. (COCl)_2_ (11.0 g, 87 mmol) was slowly added and the solution was stirred at room temperature overnight. The reaction mixture was concentrated under reduced pressure, redissolved in dichloromethane (100 mL) and cooled to 0°C. DMAP (208 mg, 1.7 mmol), Et_3_N (1.32 g, 13 mmol) and a solution of **4** (2.04 g, 10.4 mmol) in anhydrous dichloromethane (50 mL/mmol) were added successively and the resulting mixture was stirred overnight. The mixture was washed with water, dried and concentrated. The residue was purified by column chromatography (90 g silica, hexanes/EtOAc = 14:1 to 8:1) to afford **9** as a pale yellow solid (2.39 g, 57%). ^1^H NMR (200 MHz, CDCl_3_): δ = 1.25 (t, J=7.1 Hz, 3H), 2.31 (s, 3H), 3.72 (m, 2H), 3.82 (s, 3H), 3.85 (s, 3H), 6.48 (d, J=2.2 Hz, 1H), 6.62 (s, 1H), 6.66 (s, 1H), 6.94 (s, 1H), 6.97 (d, J=2.1 Hz, 1C); ^13^C NMR (50 MHz, CDCl_3_): δ = 15.0 (q, 1C), 21.4 (q, 1C), 55.9 (q, 1C), 56.7 (q, 1C), 58.8 (t, 1C), 93.1 (s, 1C), 99.2 (d, 1C), 107.1 (d, 1C), 115.7 (d, 1C), 115.9 (d, 1C), 119.6 (d, 1C), 122.0 (s, 1C), 131.9 (s, 1C), 132.2 (s, 1C), 135.4 (s, 1C), 147.9 (s, 1C), 158.6 (s, 1C), 162.1 (s, 1C), 164.7 (s, 1C); HRMS: *m*/*z* calculated: 486.0175, 509.0073 (M+Na); found: 509.0081 (M+Na).

*3,4-Bis(benzyloxy)-7,9-dimethoxy-1-methyl-6H-dibenzo[b,d]pyran-6-one (****10****)*

Compound **9** (2.27 g, 3.7 mmol), K_2_CO_3_ (0.51 g, 3.7 mmol) and Pd (OAc)_2_ (87 mg, 0.37 mmol) were suspended in DMAc (23 mL) and stirred at 120 °C (oil bath temperature) overnight. The mixture was concentrated under reduced pressure and filtered over silica (hexanes/EtOAc = 3:1 to EtOAc). During evaporation of the solvent under reduced pressure the product started crystallizing. n-Hexane was added and the pale brown precipitate was collected by filtration and washed with n-hexane to yield **10** as a pale brown solid (0.90 g, 50%). ^1^H NMR (200 MHz, CDCl_3_): δ = 2.70 (s, 3H), 3.90 (s, 3H), 3.95 (s, 3H), 5.12 (s, 2H), 5.16 (s, 2H), 6.48 (d, J=2.1 Hz, 1H), 6.66 (s, 1H), 7.17 (d, J=2.1 Hz, 1H), 7.27-7.60 (m, 10H); ^13^C NMR (50 MHz, CDCl_3_): δ = 25.6 (q, 1C), 55.6 (q, 1C), 56.4 (q, 1C), 71.0 (t, 1C), 75.7 (t, 1C), 97.4 (d, 1C), 102.7 (d, 1C), 103.6 (s, 1C), 112.3 (s, 1C), 114.1 (d, 1C), 127.4 (d, 2C), 128.0 (d, 1C), 128.1 (d, 1C), 128.3 (d, 2C), 128.7 (d, 2C), 128.9 (d, 2C), 131.0 (s, 1C), 134.0 (s, 1C), 136.7 (s, 1C), 137.5 (s, 1C), 140.6 (s, 1C), 147.2 (s, 1C), 152.0 (s, 1C), 157.2 (s, 1C), 163.9 (s, 1C), 164.7 (s, 1C); HRMS: *m*/*z* calculated: 482.1729, 483.1807 (M+H); found: 483.1816 (M+H).

*2,3-Dihydroxy-5-methylphenyl 2-iodo-4,6-dimethoxybenzoate (****11****)*

To a solution of ester **10** (2.20 g, 4.5 mmol) in MeOH (19 mL) and water (1 mL) camphor sulfonic acid (CSA) (100 mg) was added at room temperature. MeOH (10 mL) and THF (38 mL) were added and the solution was stirred overnight. To achieve full conversion, the mixture was refluxed for 4 h and then diluted with EtOAc and water. The aqueous layer was extracted with EtOAc and the combined organic layer was washed with saturated aqueous NaHCO_3_ solution and dried. The solvent was evaporated under reduced pressure to yield **11** as a pale yellow oil (1.87 g, 96%). ^1^H NMR (200 MHz, CDCl_3_): δ = 2.29 (s, 3H), 3.84 (s, 3H), 3.92 (s, 3H), 5.42 (s, 1H), 6.16 (s, 1H), 6.54 (d, J=2.1 Hz, 1H), 6.62 (s, 1H), 6.69 (s, 1H), 7.02 (d, J=2.1 Hz, 1H); ^13^C NMR (50 MHz, CDCl_3_): δ = 21.1 (q, 1C), 56.0 (q, 1C), 56.7 (q, 1C), 93.4 (s, 1C), 99.5 (d, 1C), 114.3 (d, 1C), 114.4 (d, 1C), 116.3 (d, 1C), 121.6 (s, 1C), 130.5 (s, 1C), 132.8 (s, 1C), 137.9 (s, 1C), 145.4 (s, 1C), 157.8 (s, 1C), 162.3 (s, 1C), 165.6 (s, 1C); HRMS: *m*/*z* calculated: 429.9913, 452.9811 (M+Na); found: 452.9824 (M+Na).

*2,3-Bis(benzyloxy)-5-methylphenyl 2-iodo-4,6-dimethoxybenzoate (****12****)*

To a suspension of **11** (1.77 g, 4.1 mmol) and K_2_CO_3_ (2.27 g, 16.5 mmol) in acetone (21 mL) benzyl bromide (2.11 g, 12.3 mmol) was added. The mixture was heated to 65 °C for 3 h and then stirred at room temperature overnight. After filtration the solvent was evaporated under reduced pressure. Filtration over silica (hexanes/EtOAc = 5:1) yielded ester **12** as a pale yellow solid (2.27 g, 90%). ^1^H NMR (200 MHz, CDCl_3_): δ = 2.34 (s, 3H), 3.58 (s, 3H), 3.82 (s, 3H), 5.11 (s, 4H), 6.44 (d, J=2.1 Hz, 1H), 6.74 (s, 1H), 6.83 (s, 1H), 6.87 (d, J=2.1 Hz, 1H), 7.29-7.49 (m, 10H); ^13^C NMR (50 MHz, CDCl_3_): δ = 21.6 (q, 1C), 55.9 (q, 1C), 56.0 (q, 1C), 71.2 (t, 1C), 75.0 (t, 1C), 93.2 (s, 1C), 99.1 (d, 1C), 113.2 (d, 1C), 115.5 (d, 1C), 116.1 (d, 1C), 123 (s, 1C), 127.6 (d, 2C), 127.7 (d, 1C), 128.1 (d, 2C), 128.3 (d, 2C), 128.5 (d, 1C), 128.6 (d, 2C), 133.9 (s, 2C), 137.0 (s, 1C), 138.1 (s, 1C), 144.3 (s, 1C), 152.9 (s, 1C), 158.5 (s, 1C), 162.0 (s, 1C), 165.9 (s, 1C); HRMS: *m*/*z* calculated: 610.0852, 633.0745 (M+Na); found: 633.0755 (M+Na).

***4-OH-AOH*** *and* ***4-OH-AME***

To a solution of precursor **12** (290 mg, 0.60 mmol) in anhydrous dichloromethane (60 mL) BBr_3_ (1.02 mL, 10.8 mmol) was slowly added at -80 °C. After complete addition the solution was stirred at -80 °C for 30 min and then stirred at room temperature for 3 d. The mixture was quenched by careful addition of MeOH at 0 °C and applied to silica (1 g). Purification by column chromatography (90 g SiO_2_, 100% dichloromethane to dichloromethane/MeOH = 15:1) yielded both crude products. Further purification was done by reversed phase chromatography (8g RP-C18, MeOH/water = 1:1) and gave **4-OH-AME** (104 mg, 60%) and **4-OH-AOH** (33 mg, 20%) as pale yellow solids.

4-OH-AOH: ^1^H NMR (400 MHz, CDCl_3_): δ = 2.63 (s, 3H), 6.34 (s, 1H), 6.71 (s, 1H), 7.24 (s, 1H), 9.10 (s, 1H), 9.87 (s, 1H), 10.86 (s, 1H), 11.86 (s, 1H); ^13^C NMR (100 MHz, CDCl_3_): δ = 24.8 (q, 1C), 97.3 (s, 1C), 100.9 (d, 1C), 104.4 (d, 1C), 109.3 (s, 1C), 116.8 (d, 1C), 126.3 (s, 1C), 131.2 (s, 1C), 138.7 (s, 1C), 141.5 (s, 1C), 146.8 (s, 1C), 164.1 (s, 1C), 164.6 (s, 1C), 165.4 (s, 1C); HRMS: *m*/*z* calculated: 274.0477, 275.0555 (M+H); found: 275.0560 (M+H).

4-OH-AME: ^1^H NMR (400 MHz, DMSO-d_6_): δ = 2.65 (s, 3H), 3.90 (s, 3H), 6.61 (s, 1H), 6.72 (s, 1H), 7.22 (s, 1H); ^13^C NMR (100 MHz, DMSO-d_6_): δ = 24.5 (q, 1C), 55.8 (q, 1C), 98.3 (s, 1C), 99.2 (d, 1C), 103.4 (d, 1C), 109.1 (s, 1C), 116.9 (d, 1C), 126.4 (s, 1C), 131.2 (s, 1C), 138.4 (s, 1C), 141.5 (s, 1C), 147.0 (s, 1C), 164.1 (s, 1C), 164.6 (s, 1C), 166.1 (s, 1C); HRMS: *m*/*z* calculated: 288.0634, 289.0712 (M+H); found: 289.0721 (M+H).

Semi-preparative HPLC and solid phase extraction

To reach a high purity of both hydroxylated metabolites, a semi preparative HPLC method followed by a subsequent solid phase extraction was applied. Therefore the purification was carried out using a Knauer Smartline 1000 (LPG) pump and an Agilent 1200 series G1315D UV/VIS DAD with the software Clarity Chrom. Separation was done on a semi-preparative Phenomenex Luna 5u C18 (2) column (21.2 mm (i.d.) × 250 mm, 100A) with the mobile phases bidest. H_2_O (solvent A) and methanol (solvent B). The gradient program (1 min 45% B; 3 min 50% B; 6 min 60% B, 15 min 100% B; 20 min 100% B) and the flow rate (10 mL/min) were set and fractions were collected by hand after 16 min for 4-OH-AOH and 20 min for 4-OH-AME at the detector outlet. The solutions were evaporated to dryness. After separation, cleaning by solid phase extraction (SPE) with LiChrolut® RP-18 (40-63 µm; Merck, Darmstadt) was executed. At first, the columns were conditioned with 6 mL ACN, followed by 6 mL bidest. H_2_O. The substances were dissolved in CAN H_2_O (50:50, v/v) and applicated on the columns. After infiltration, the columns were rinsed with 4 mL H_2_O and each 2 mL 10, 20, 30 and 40% ACN. The 50% ACN fraction (4 mL) was collected, evaporated to dryness and weighted. The chromatographic purity was detected with HPLC at 280 nm and was about >95%.

**Online Resource 2**

**Fig. S1** Selected reaction monitoring (SRM)-chromatograms of AOH, AME, 4-OH-AOH and 4-OH-AME

**Online Resource 3**

**Fig. S2** Selected reaction monitoring (SRM)-chromatograms of 4-OH-AOH and an additional peak present after 1h incubation at *m/z* 287→272 which might correspond to a methylation product
